# Supplementary material for: Are carbon emissions trading and green financial instruments synergistic? -Comprehensive quantitative research based on content analysis
Source: PLoS One. 2024 Mar 7;19(3):e0298601. doi: 10.1371/journal.pone.0298601 (PMC10919601; doi:10.1371/journal.pone.0298601)
Supplement: S1 Data — (ZIP) [file pone.0298601.s001.zip › Data/地级市Z_F工作报告.zip!/║╙─╧/╞╜╢Ñ╔╜╩╨/└┤╫╘╣1⁄2╓┌║┼í╛┬φ┐╦╩2╛▌═°í┐_2006.docx]

各位代表:

现在,我代表市人民政府,向大会作政府工作报告,请予审议,并请政协委员们提出意见。

一、2005年及“十五”工作回顾

2005年,市人民政府在省委、省政府和市委的正确领导下,认真落实科学发展观,努力构建和谐社会,全面完成了市八届人大二次会议确定的各项任务。

2005年的工作成效集中表现在:国民经济快速增长、综合效益明显提高、发展活力不断增强、社会事业全面进步、省定目标全部完成、经济目标超额完成。全市生产总值初步核算558.3亿元,比上年增长15.3%,高出全省1.2个百分点;地方财政一般预算收入29.1亿元,增长48.8%,增速居全省第1位;全社会固定资产投资190.4亿元,增长67.3%,其中城镇固定资产投资155.2亿元,增长63.3%;社会消费品零售总额146亿元,增长14.4%;外贸出口2.1亿美元,增长69%,增速居全省第1位;金融机构各项存款余额493.4亿元,贷款余额251亿元,分别比年初增长22.2%和25.2%,增速均居全省第1位;城镇新增就业7.5万人,超过预期目标;城镇居民人均可支配收入8723元,农民人均纯收入2688元,扣除价格因素,分别增长11.9%和11.1%。我市在全面建设小康社会道路上又迈出了坚实的一步。

2005年,我们主要做了以下工作:

(一)狠抓重点项目建设。我们紧紧抓住新一轮经济增长机遇,在煤炭电力、农林水利、生态环保、基础设施、社会事业等领域,开工和建成了一批重点项目,保持了投资对经济增长的强劲拉动。针对项目建设中存在的问题,在落实项目法人负责制基础上,实行了市级领导联系重点项目和对重点项目建设进度的督察制度,帮助协调资金和环境问题,有力促进了重点项目建设。年初安排的重点项目共完成投资90.9亿元,占全年预期投资的162.3%。建成了天瑞集团日产5000吨水泥及铁路高速重载机车铸钢件、中加公司50万吨炼铁、中盐皓龙60万吨精制盐、平临高速临汝至市区段等20个项目;续建和新开工了37个项目,其中神马尼龙66盐10-20万吨改扩建、2万吨连续聚合尼龙66切片、5万吨离子膜烧碱,220千伏宝丰至汝州王寨输电项目,省城市天然气管网平顶山子项目,市污水处理二期等项目提前建成。同时,认真谋划长远发展,完成了我市“十一五”发展总体规划及专项规划的研究、编制工作,筛选了一批重点建设项目,为今后五年持续快速健康发展奠定了基础。

(二)引导产业结构优化。一是强化工业主导。在稳定发展煤炭产业的同时,引导新上了一批火力发电、化工化纤、钢铁水泥、机电制造、井盐加工、盐化工、纺织等非煤产业项目,工业对经济增长的支撑作用明显增强。全市规模以上工业实现增加值200.1亿元,增长23.6%;利润37.1亿元,增长96%;综合效益指数158.7%,提高30.3个百分点。工业对经济增长的贡献率达64.8%,拉动经济增长9.9个百分点。二是加强农业基础。全市财政支农支出3.2亿元,增长25.5%。支持了汝州巨龙淀粉、梦想食品扩建和鲁山张良腰带出口,市级以上龙头企业发展到41家。推广了叶县农民专业合作经济组织经验,全市农民专业合作经济组织新增54家,达到130家。推进了农业标准化生产,省定无公害农产品产地新增22家,达到58家;无公害畜产品产地新增23家,达到49家。扶持了宝丰、郏县、湛河区奶牛和乳业加工基地,舞钢肉鸡、叶县肉牛、宝丰生猪基地,全市畜牧业产值占农林牧渔业比重49.5%,提高0.9个百分点。全年造林11.3万亩,其中经济林1.8万亩。三是提升服务业层次。在努力发展传统服务业的同时,更加注重发展现代服务业和新兴服务业,特别是文化产业和旅游产业。宝丰县大力发展文化产业的经验,被中宣部、文化部誉为“宝丰文化现象”,并在全国推广。全市文化及相关产业总产出48.6亿元,增长17.1%。继续加强景区配套设施建设,新开工和续建旅游项目19个,完成投资3.3亿元。旅游业总收入10.6亿元,接待游客246.4万人次,分别增长22.4%和23.2%。房地产、金融、保险、信息等服务业也都呈现加快发展势头。

(三)放手发展县域经济。通过县(市、区)经济发展现场观摩和经验交流,组织各县(市、区)主要负责同志到省内先进市县考察学习,各县(市、区)的发展思路更加明确,重点更加突出,成效更加显著。6县(市)实现生产总值358.6亿元,增长17.5%,占全市生产总值的比重提高到64.2%;10个县(市、区)财政一般预算收入全部超亿元,其中6县(市)财政一般预算收入11.6亿元,增长63.3%,占全市的比重提高到39.8%。县域主要经济指标增速均高于全市平均水平。汝州天瑞集团、鲁山汇源公司、舞钢中加公司、宝丰洁石建材集团等一批民营骨干企业进一步壮大,为县域经济注入了活力。全年培训农民工12万人,新转移农村富余劳动力20万人,汝州、舞钢、鲁山被确定为省级劳务输出基地。全年实现劳务收入57亿元。根据第五届全国县域经济基本竞争力评价结果,汝州市和舞钢市位次分别比上届提升35位和282位;在中部百强县(市)中,分别排第30位和第50位。

(四)积极推进改革开放。针对我市国有企业改制中存在的问题,出台了《关于进一步规范国有企业改制工作的实施意见》和《平顶山市改制企业职工安置补偿办法》,规范了改制行为,加大了改制力度,新完成国有企业改制45家。平煤集团33所中小学全部移交政府管理。国有粮食购销企业由112家改制重组为34家,分流安置富余职工5533人,多元化粮食市场体系基本形成。全市10家农村信用联社全部取得了专项央行票据。深化了乡镇机构改革,全市撤并乡镇11个,分流、清退7291人。交通、水利、科技、教育、文化、卫生等方面的改革也都取得新的进展。搭建了政银企合作平台,实施中小企业创业辅导和融资信用担保等扶持措施,促进了非公有制经济发展。全年非公有制经济实现增加值231.7亿元,增长15.8%,占全市生产总值的41.5%,提高2个百分点。组团参加和举办了一系列招商引资活动,对外开放取得新成效。全年新批外商投资企业11家,实际利用外商直接投资4368万美元,增长26.9%,利用市外资金46亿元,增长25%。

(五)着力优化发展环境。以创建中国优秀旅游城市为契机,完成了市政大厦、高阳路二期、体育路、劳动路、大乌路等重点市政工程。强化城市三区的建设和管理责任,改造了24条背街小巷,新建了一些街头游园,城区增加绿地14万平方米,植树119.3万株,城市日常管理和环境面貌有了新变化。新城区开工建设项目42个,总投资36亿元,年度完成投资15亿元,起步区10平方公里基础设施配套到位,行政中心和湖滨路中段景观带已具雏形,“三年出形象”的目标基本实现。县城建设和管理有了新起色。以转变政府职能、提高行政效能为重点,继续深化行政审批制度改革,进中心的500个行政审批事项平均办理时间,由上年的12.4个工作日进一步压缩到5.2个工作日。查处“四乱”等破坏经济发展环境案件101起。在全省率先推行为民服务全程代理制,全市共设置乡级代理中心137个,村级代办点2573个,初步形成了四级便民服务网络。

(六)努力构建和谐社会。一是更加重视发展社会事业。投入4043万元,安排科技项目341个,又有14家企业、17种产品分别被认定为省级高新技术企业和高新技术产品;支持了农科所农业生物技术等重点实验室建设;全年技术专利申请234项,增长56%。筹措资金5294万元,消除D级危房15.4万平方米,提前一年完成了省定任务;发放资金4933万元,全面落实了农村义务教育“两免一补”政策。筹措资金5131万元,建成了7个疾病控制中心、8个传染病医院(病区)和市紧急救援中心,改造了15个乡镇卫生院;在郏县开展了新型农村合作医疗试点,参合农民达到32.5万人;卫东区荣获“全国社区卫生服务示范区”称号。继续推进计划生育综合改革,认真落实计划生育家庭奖励扶助政策,稳定了低生育水平,全市人口自然增长率5.1‰,低于省定目标。文化精品工程取得新的成果。我市豫剧团演出的《珠帘秀》、曲剧团演出的《明月芳魂》分别获得我省首届文华奖、文华新剧目奖,《明月芳魂》还获省“五个一工程奖”。市文化艺术学校、宝丰县分别被评为全国文化工作先进单位和全国文化市场管理先进单位。投资345.6万元,完成了50户以上自然村广播电视“村村通”工程。举办了第九届全民健身节,完成了第二次国民体质测试,竞技体育水平也有新的提高。二是更加重视安全生产和环境保护。认真执行安全生产的各项法律法规,加大矿产资源整合力度,严格落实安全生产责任,依法加强监督检查,保持了安全生产形势总体平稳。全市关闭小煤矿243座,井筒442个;关闭非煤矿山80座,危险化学品经营单位30家。全年各类伤亡事故起数、死亡人数同比分别下降45%和13.6%,其中煤矿事故起数、死亡人数分别下降92.8%和95.5%,煤矿百万吨死亡率0.26,创历史最好水平;亿元产值死亡率、万人死亡率均低于省定控制指标。充分运用经济、法律和行政手段,对重点排污企业、焦化行业以及小炼焦、扬尘污染、秸秆焚烧等进行了综合执法,依法关闭淘汰了115家高污染企业,城市大气质量达标224天,全面完成了省定环保目标。三是更加重视民生。全年新增就业岗位7.5万个,安排下岗失业人员再就业3.4万人,其中“4050”人员8934人。筹措和发放下岗职工基本生活费3907万元,发放小额担保贷款7670万元。征集养老、失业、医疗、工伤、女工生育保险金8.8亿元,支付各项社会保险金7.3亿元。发放城市居民最低生活保障金5410万元,8.1万城市居民得到低保救助。全市取消了农业税,农民减负8038万元;发放粮食直补、良种补贴和大型农机具补贴4886万元。投资3.2亿元用于农村公路建设,农村“村村通”新解决389个行政村。投资1.6亿元,完成了县城电网年度改造任务。投资309万元解决了重污染地区1.4万人的饮水安全问题。完成了134个村的整村推进扶贫任务,又有4.3万贫困人口和低收入人口脱贫。发放农村特困户救助资金1000多万元,“五保”供养资金2000多万元,9.6万农村人口得到救助。投资1191万元,完成倒房重建1800多间,受灾群众全部搬进新居。投资1000万元,建成了市夕阳红老年公寓。爱心救助超市救助困难群众1.7万人次。市委、市政府承诺的12件实事全部落实。四是更加重视精神文明和民主法制建设。深入开展了创建文明城市、文明景区和“道德规范进万家、诚实守信万人行”等活动,不断加强未成年人思想道德建设和大学生思想政治教育,城乡文明程度不断提高。舞钢市创建成为中国优秀旅游城市。市燃气总公司和市地税局被命名为全国文明单位。继续开展联村帮扶工作。顺利完成了第五届村委会换届选举,村级基层组织建设得到进一步加强。严格执行人大及其常委会通过的决议和决定,自觉接受人大法律、工作监督和政协民主监督,办理人大代表建议和政协提案401件,办复率100%。认真开展全民普法教育,积极推进创建“民主法治示范村”、“法律进社区”等活动,“四五”普法和“二五”依法治市工作顺利通过验收。坚持依法行政,政府工作法制化水平进一步提高。廉政建设和反腐败斗争取得新成效,全年共查处各类违纪违法案件789件,政纪处分378人。军政军民团结更加巩固,第三次荣获全省“双拥模范城”称号。加强社会治安综合治理,组织开展了“命案侦破攻坚战”及打击和预防“两抢一盗”等专项斗争,刑事发案量下降13.6%。认真做好群众来信来访工作,加强市长热线网络建设,积极化解人民内部矛盾。制定完善了各类应急预案,处理突发事件能力不断增强。市史志办被评为全国地方史志工作先进单位。民族宗教、统计、审计、外事、侨务、档案、对台、人防、气象、物价、盐业、新闻出版、国家安全等各项工作都取得了新的成绩。

2005年各项目标的全面完成,使“十五”计划的大部分指标超额完成。经济实力明显增强。生产总值五年新增286.8亿元,年均增长11.7%,高出计划3.7个百分点,高出全省0.3个百分点,实现了“十五”期间赶上或超过全省平均水平的奋斗目标。人均生产总值突破1万元。综合效益显著提高。地方财政一般预算收入是“九五”末的2.7倍;规模以上工业综合效益指数比“九五”末提高80.6个百分点。投资力度不断加大。五年完成全社会固定资产投资496.2亿元,是“九五”时期的1.9倍。高速公路实现“零”的突破,通车里程达158公里。人民生活继续改善。城镇居民人均可支配收入、农民人均纯收入年均增长12.7%和6.4%;社会消费品零售总额比“九五”末增加61.5亿元;城乡居民储蓄存款余额326.8亿元,比“九五”末增加184.9亿元;城镇人均住宅建筑面积23.4平方米,比“九五”末增加3平方米;新解决农村饮水困难36.8万人。社会事业发展加快。科技进步对经济增长的贡献率达到45%;平顶山学院、平顶山工学院实现专升本,普通高校在校生达3.4万人,是“九五”末的4.8倍;五年平均人口自然增长率5.3‰,低于全省平均水平。我市经济社会发展已经站在了新的战略起点,进入了全面提升产业层次、加快经济转型、促进统筹协调发展的新阶段。

回顾去年及“十五”的工作,我们主要有以下深刻体会:一是必须坚持科学发展观,坚定不移地推进“实施二次创业，打造经济强市”,建设新型能源和工业基地的发展战略;二是必须坚持围绕全面建设小康社会目标,持之以恒地加快工业化、城镇化,推进农业现代化;三是必须坚持扩大固定资产投资,不断增强经济发展后劲;四是必须坚持改革开放,不断增强发展的内在动力与活力;五是必须坚持优化发展环境,不断增强城市竞争力;六是必须坚持以人为本,着力解决涉及人民群众切身利益的突出问题。

各位代表,这些成绩的取得,是省委、省政府和市委正确领导的结果,是人大、政协积极支持的结果,是全市人民共同奋斗的结果。在此,我代表市人民政府,向工作在全市各条战线上的工人、农民、知识分子、干部、人民解放军驻平部队指战员、武警消防官兵和公安民警致以崇高敬意!向关心、支持我市现代化建设的各界人士表示衷心感谢!

我们也清醒地看到,我市与先进市差距仍然较大,经济社会发展仍存在一些突出矛盾和问题:经济快速增长主要依赖生产要素的高投入和资源的高消耗,经济发展与人口资源环境的矛盾日益加剧,结构调整和经济增长方式转变的任务仍然很重;农民收入增长缓慢,城乡居民收入差距拉大;部分领域改革滞后;经济外向度低;企业自主创新能力不强,高新技术产业比重小;银行存贷差仍然较大;一些群众的生产生活还比较困难,关系群众切身利益的就业、就医、子女上学等问题仍较突出;安全生产形势依然严峻;城市建设与管理滞后;经济发展环境还不宽松;政府的社会管理和公共服务职能还比较薄弱;一些部门之间职责不清,协调不力,管理方式落后,办事效率不高;形式主义、官僚主义和奢侈浪费的行为时有表现;腐败现象在一些地方、部门和单位仍有发生。对以上问题,我们要高度重视,并在今后工作中认真加以解决。

二、“十一五”发展的基本思路和奋斗目标

“十一五”时期,是我市提前实现全面建设小康社会承前启后的关键时期,也是人均生产总值突破1000美元后,经济社会发展的“战略机遇期”和“矛盾凸显期”。抓住机遇,迎接挑战,使我市在“十一五”时期经济社会快速协调发展,是我们面临的重大任务。根据市委六届六次全会提出的“十一五”发展指导思想,《平顶山市国民经济和社会发展第十一个五年规划纲要(草案)》(以下简称《纲要(草案)》)提出了今后五年经济社会发展的基本思路和奋斗目标。

基本思路是:坚持以邓小平理论和“三个代表”重要思想为指导,以科学发展观统领经济社会发展全局,以提高经济增长质量和效益为中心,坚定不移地推进“实施二次创业，打造经济强市”,建设新型能源和工业基地的发展战略。以技术创新为重点,转变增长方式,科学有序地开发利用资源,着力促进工业结构优化升级;以农民增收为重点,提高农业产业化水平,促进农村劳动力转移,着力建设社会主义新农村;以增强城镇辐射能力为重点,加快实施中心城市带动战略,加强城镇基础设施建设,着力提升城镇化水平;以优化经济发展环境为重点,深化改革开放,转变政府职能,着力提高行政效能和经济市场化水平;以解决人民群众最关心、最直接、最现实的利益问题为重点,全面发展各项社会事业,加强环境保护和安全生产,着力构建和谐社会,努力开创社会主义经济、政治、文化、社会建设新局面。

奋斗目标是:在更加重视质量和效益的前提下,努力实现经济总量和财政收入“双翻番”。经济实力明显增强,产业层次明显提高,城镇化进程明显加快,人民生活明显改善,生态环境明显好转,社会事业明显进步。生产总值年均增长12%以上,2010年达到1100亿元以上;地方财政一般预算收入年均增长12%以上,2010年达到60亿元;城镇化率年均提高2.6个百分点,2010年达到48%以上;单位生产总值能耗比“十五”末降低20%左右。经过五年和更长一段时期的努力,把平顶山建设成为国家重要的能源和原材料生产加工基地,中国中部化工城;以化学工业、装备制造工业为战略走向的新型工业城市;国家优秀山水文化旅游城市。

为实现上述目标,《纲要(草案)》还提出了今后五年的主要任务。基本思路、奋斗目标和主要任务是在正确把握国际国内形势,认真研究国家宏观经济政策,全面分析我市各种有利条件,深入查找存在问题与差距的基础上提出的。这些目标对于加速我市经济社会发展不仅是必需的,而且通过努力也是完全可以实现的。《纲要(草案)》及说明已印发各位代表,请予审议。

三、2006年的主要任务

今年是“十一五”发展的开局之年。我市经济社会发展的主要预期目标是:全市生产总值增长12%以上;地方财政一般预算收入增长12%以上;全社会固定资产投资增长20%以上;社会消费品零售总额增长15%以上;外贸出口增长15%以上,实际利用外资增长25%以上;城镇居民人均可支配收入增长10%左右,农民人均纯收入增长8%左右;居民消费价格总水平涨幅控制在3%以内;单位生产总值能耗降低4%左右;人口自然增长率控制在6‰以下;城镇登记失业率控制在4.5%以内,新增就业6万人。

围绕上述目标,重点抓好十个方面的工作:

(一)继续加大固定资产投资和重点项目建设力度。今年,全市共安排500万元以上的固定资产投资项目273个,总投资938亿元,当年预期完成投资186亿元。其中竣工项目121个,年度投资50亿元;续建项目45个,年度投资104亿元;新开工项目75个,年度投资32亿元;前期项目32个,总投资441.5亿元。今年确定的第一批重点项目87个,总投资627亿元,年度投资109亿元。其中,燕山水库、农村安全饮水等农业项目7个,年度投资3亿元;舞钢公司100万吨中厚板、平煤集团瑞平公司日产2×5000吨干法水泥生产线等工业项目33个,年度投资45.3亿元;郑石高速、太澳高速平顶山段,姚电公司2×60万千瓦扩建,程平路改造等交通、能源和城市基础设施项目36个,年度投资59.4亿元。要认真落实项目法人负责制度、市级领导联系重点项目制度和工程督察制度,确保重点项目建设进度。

(二)扎实推进社会主义新农村建设。目前,“三农”问题的主要矛盾仍然是农村经济发展不快,农业生产力水平较低,农民收入增长缓慢,农村面貌落后。因此,今年“三农”工作的重点是,坚持以发展农村经济为中心任务,全面推进社会主义新农村建设。加大对“三农”的投入力度。筹措资金3586万元,对种粮农民进行直接补贴,并继续实行良种补贴和购买大型农机具补贴,稳定粮食生产;扶持发展蔬菜、烟叶、食用菌、花卉、瓜果等高效经济作物生产和以速生丰产林为主的经济林生产。筹措资金4695万元,对退耕还林农户进行补助。筹措资金2000万元,用于农村扶贫开发,解决4.3万农村贫困人口的脱贫和温饱问题。多方筹措资金,加大工作力度,完成全市所有行政村通水泥路(或油路)的建设任务,并改建县乡道路57公里。建立和完善以县为主的农村公路养护管理体制。筹措资金2400万元扶持沼气建设,新建沼气池3万座。筹措资金1440万元,解决高氟、高盐及污染地区4万农民安全饮水问题。筹措资金342万元,解决部分深山散居农民的迁居问题。抓好昭平台灌区节水和龙兴寺水库除险加固等工程建设,做好南水北调中线工程的前期准备工作。发挥国家农业资金投入的导向作用,鼓励社会资本积极参与农业和农村基础设施建设。加大对农业产业化的培育力度。按照市场牵龙头、龙头带基地、基地连农户的农业产业化链条,认真落实财政补贴、贷款担保等优惠政策,扶持农业产业化龙头企业,带动农业结构优化,增加农民收入。重点培育巨龙淀粉、梦想食品、华懋双汇、正大饲料、万东牧业等一批规模较大、带动作用较强的龙头企业,力争今年再有3家进入省级重点扶持企业。加快发展畜牧业,新建规模养殖场205个,养殖小区28个,新增奶牛1万头,完成肉产品产地认证15个,畜牧业产值比重达到50%以上。提高农业组织化水平,全年新增农民专业合作经济组织50家以上。建立健全农产品检验检测、市场准入、质量追溯、认证、标识和公示制度。高度重视禽流感等重大动物疫病防控工作,全面完成县级动物防疫体系建设任务。加大对农村劳动力培训和转移的引导力度。继续实施“农村百万劳动力转移工程”,筹措资金7784万元,完成40万农民的科技培训及14万农民的职业技能培训,新转移农村富余劳动力10万人以上。继续深化农村改革。巩固乡镇机构改革成果,确保乡镇机关事业人员只减不增。转变乡镇政府职能,提高为“三农”服务的水平。深化农村义务教育和县乡财政体制等改革。进一步提升县域经济发展水平。重点加强县城和小城镇基础设施建设,完善公共服务功能;大力发展特色产业,提高县域经济的集中度和关联度;搞好各县(市)的工业园区建设,提升县域工业的质量和效益,加快县(市)工业化发展步伐。切实用足用活中央、省、市支持县域经济发展的各项政策,创造性地开展工作,力争县域经济在全省综合经济实力排序中位次前移。积极探索建设社会主义新农村的有效途径。建设社会主义新农村,是加强“三农”工作的重大举措,同时又是一个全面的目标,是一项长期的任务。必须按照“生产发展、生活宽裕、乡风文明、村容整洁、管理民主”的要求,科学规划,因地制宜,分类指导,有步骤有计划有重点地积极推进。加大工业反哺农业、城市支持农村力度,开展市县乡机关和重点企业“联村帮建”活动。各部门要相互配合,加强对各方面涉农资金的协调整合,注重使用的综合效益。市里重点选择10个行政村，每个县(市、区)也要选择3~5个行政村进行重点“帮建”。通过典型带动,建设一批社会主义新农村示范村。

(三)加快培育新型能源和工业基地。着力发展新兴产业。今年我市培育新兴产业的标志性项目是启动中国中部化工城(园区)建设。园区选址论证和各项前期准备工作要尽快完成,平煤、神马已落实的项目要抓紧开工,确保年内取得实质性进展。同时,开工建设平高集团特高压组合电器产业化项目、汇源公司20万吨烧碱和20万吨PVC树脂项目;加快汝州鸿瑞煤化、石龙区鸿跃公司二甲醚和汝州梨园1亿标块煤矸石空心砖、宝丰三桩公司粉煤灰利用等项目建设;建成汇源公司“双十氯碱”、鲁山强生生物制药、郏县荣生堂生物制药、圣光公司新厂区、中盐皓龙天源60万吨精制盐等项目。做大做强传统优势产业。年内建成平煤集团瑞平公司张村矿。姚电公司2×60万千瓦机组完成投资11亿元,平顶山电厂2×20万千瓦热电联产技改项目上半年建成投产,蓝光电厂一期工程二号机组完成主机安装,郑营变电站、蒲楼变电站力争2月底投入运行,双山180兆伏安输变电项目完成土建工程,开工建设鲁山变电站、王寨变电站扩建等电网项目。抓紧做好平顶山电厂二厂一期、姚电公司五期扩建2×100万千瓦机组、500千伏安超高压平西变电站等项目前期工作。舞钢公司100万吨中厚板生产线年内试运行;平煤集团瑞平公司日产2×5000吨、天广集团日产5000吨、天瑞集团日产4000吨、宝丰大地公司日产5000吨干法水泥生产线年内完成土建工程,力争建成投产。神马集团6000吨高模低缩涤纶工业丝和2万吨特品工业丝项目分别在6月份和10月份之前建成投产。不断推进技术创新。企业是创新的主体,鼓励企业增强原始创新、集成创新和引进消化吸收再创新能力,运用新技术、新工艺、新科技成果改造传统产业,淘汰落后生产能力,提升产业整体技术水平,争取全年有30个项目通过省级以上科技成果鉴定。依托平煤、神马等企业,加大与大专院校、科研院所的联合攻关力度,围绕煤盐化工、新材料等领域,开发研制出核心技术、独特工艺和关键设备;依托平高集团、舞钢公司,提升超高压电器、特宽厚钢板研发中心的技术创新能力;依托姚电公司、中材环保公司、天瑞集团,推动火力发电、环保设备、铸钢配件技术研发中心建设。通过科技创新,打造一批在国内外有影响力的知名品牌。培育壮大企业集团。市里安排一定的配套资金,积极争取省工业结构调整和高新技术产业化资金,以及国债资金和银行贷款,综合运用各项改革发展措施,推动市30户重点企业和10户企业集团进一步做大做强。加强工业经济运行调节。搞好电力、煤炭、运力的供求衔接。加强银企沟通,引导和协调银行资金向市场前景好、经济效益高的企业流动。对亏损企业分类指导,促其兼并重组、强化管理、创新技术,提高经济效益。

(四)进一步提升城镇化水平。今年中心城市、卫星城和小城镇建设的重点是,强化规划和规划执法、加大投资力度、提高管理水平、畅通城市交通、改善环境质量、完成“创优”任务。突出抓好中心城市建设。新城区建设按照“五年成规模”的目标要求,继续加大建设力度,今年各类新建、续建项目总投资40亿元,年度投资18亿元,其中基础设施投资5亿元。重点抓好各进驻单位办公楼、会展中心、住宅小区建设,湖滨路东西延伸、纬一路向西拓宽,以及管网、医院、学校等配套工程。筹措资金6亿元,用于老城区改造。年内完成程平路、建设路、矿工路、高阳路、东风路、园林路改造,新华路南北延、开源路南延、光明路北延、凌云路及河东路湛河桥、开发一路湛河桥、开发二路湛河桥连线等工程建设。同时,区属27条背街小巷全部完成改造。按照创建“中国优秀旅游城市”要求,新建10座垃圾中转站、30座星级公厕、10座普通公厕,改造54座水冲式公厕。建成和平路商业步行街。联盟路连片开发主体完工。积极推进大营、铁炉、西高皇等“城中村”改造,火车站广场改造,东立交桥绿化,继续进行湛河治理、湛河三期绿化和湛河亮化,进一步搞好建设路两侧绿化,公园、游园、广场绿化等生态、公益工程。城市建成区绿地率由上年的24.9%提高到27%以上,绿化覆盖率由29.1%提高到32%以上。努力改善城镇面貌。县城要强化规划管理,着重抓好城区道路及水、电、通信、绿化、排污、污水处理、垃圾处理、天然气利用等基础设施建设,完成县城电网改造。以建制镇建设为重点,指导乡镇政府搞好城镇规划,盘活土地资产,加快建设进度。到年底,乡镇政府所在地的面貌要发生明显变化。支持舞钢市搞好城乡一体化试点。切实加大城市管理力度。进一步坚持和完善“两级政府,四级管理”体制,建立健全各级城管责任制。全力整治市容环境卫生,着力解决占道经营、乱贴乱画、交通拥堵,特别是背街小巷、城乡接合部脏乱差等人民群众反映强烈的问题,搞好城市绿化、美化、亮化。加强社区建设,完善社区服务功能。加快发展服务业。重点是大力发展文化产业和旅游产业。加大文化和旅游资源的整合力度,充分挖掘并合理利用人文资源和温泉资源。进一步加强旅游精品线路和石人山、石漫滩、昭平湖等重点景区基础设施建设,提高服务质量,搞好宣传推介,开拓旅游市场,全面提升旅游业发展水平,全年旅游业总收入增长15%以上。总结推广宝丰发展文化产业的经验,加快文化体制改革步伐,发展壮大文化产业,全年文化及相关产业总产出增长17%以上。搞好物流业资源调查和发展规划,促进物流产业上水平。加快商贸中心、步行街、特色街以及新城区综合市场、农贸市场建设。积极发展农村商业网点和连锁经营,加快农村市场体系建设。筹措资金6000万元,更新公交大巴车150台、出租车300辆,提升交通服务业水平和城市形象。进一步促进房地产、金融、保险、信息等其他服务业的发展。

(五)积极建设资源节约型和环境友好型社会。加快发展循环经济。支持平煤集团抓住被列为全国循环经济试点企业的机遇,充分利用国家的配套政策,大力发展接续产品,拉长产业链条,发展循环经济。支持神马集团加大工作力度,力争年内进入国家第二批循环经济试点企业。支持全市各类企业认真落实国家出台的有关政策,搞好本企业循环生产,特别是搞好煤矸石、粉煤灰、电石渣、生产生活垃圾的综合开发利用,推进中水回用、机焦废气回收和瓦斯利用。支持农作物秸秆等生物质能源和生物质材料的资源化利用。固体废弃物综合利用率达到66%以上。大力推进资源节约。加大对高耗能、高耗材、高耗水行业的管控力度。开发利用节能、节水、节地、节材等节约技术,力争规模以上工业万元增加值耗水由上年的540吨减少到510吨,耗电由3770度减少到3660度。全面清理整治砖瓦窑厂,扶持发展新型墙体材料,关闭实心黏土砖瓦窑118座。进一步盘活存量建设用地,提高土地集约利用水平。继续整顿和规范矿产资源开发秩序。加强环境保护和生态建设。加大对城市规划区内污染企业的改造、搬迁和关闭力度,对治理力度大、投入资金多的实行奖励,不能按时达标的进行处罚。新上项目必须科学布局,严格通过环保评审,严格执行“三同时”(环境保护设施与主体工程同时设计、同时施工、同时投产使用)。年度投资2.5亿元,对采矿沉陷区进行综合治理。关闭12条生产工艺落后、环境污染较大的水泥生产线,对22家耐火材料厂、12家碳素厂进行整顿,巩固焦化行业治理成果,市区及周边的所有电厂烟气脱硫除尘必须达标。继续抓好秸秆禁烧和综合利用工作。城市大气质量优良天数达到256天。加大水污染防治力度,平西湖水质保持三类标准。各县(市)的污水处理项目建设,必须按计划如期完成。巩固造林绿化成果,全面完成今年的林业重点工程,不断改善生态环境。

(六)坚持深化改革开放。进一步推进各项改革。今年国有企业改制要基本完成,经营性事业单位改制大部分完成,建设系统三项改革全面完成。供销体制和粮食流通体制改革继续巩固完善。安排专项资金3000万元,用于市属国有企业改制中职工的有关补助。进一步完善部门预算管理体系,加强行政事业单位国有资产管理,加大政府采购力度。制定出台《金融生态环境监督考评办法》,进一步加强银企合作,创造金融生态区。支持城市信用社加大改革力度,组建商业银行。大力发展非公有制经济。认真落实促进非公有制经济发展的有关政策措施,进一步放宽市场准入,优化发展环境。建立健全中小企业融资担保、创业辅导等社会化服务体系。继续扩大对外开放。落实出口退税政策,优化出口商品结构,提高出口商品竞争力,全年外贸出口实现2.4亿美元。筹划举办两次省外招商会,组织参加国家和省举办的重点招商引资活动。积极推行网上招商、中介机构招商、委托招商等市场化招商形式。强化企业招商的主体地位。加强签约项目跟踪落实,提高招商实效。全年直接利用外资5460万美元以上。继续修订完善招商引资优惠政策,严肃查处破坏经济发展环境的案件,营造安商、亲商、护商的良好氛围,增强环境的吸引力。

(七)全面发展各项社会事业。大力发展科技事业。把提高自主创新能力摆在全部科技工作的首位,努力开发新能源、新材料和环保新技术,多方筹措资金,支持科技成果转化和专利应用,着力解决制约经济社会发展的重大科技问题。依法保护知识产权。继续实施“科技富民工程”,完善农村科技服务体系。大力实施人才强市战略,完善人才流动政策,使优秀人才能够脱颖而出,施展才干。全面发展教育事业。筹措资金4750万元,对农村义务教育阶段贫困家庭学生继续实行“两免一补”。确保农村中小学公用经费的落实。建立农村中小学D级危房改造长效机制。加快实施农村中小学远程教育工程。大力发展职业教育,培养社会急需的各类技能型人才。搞好规划,创造条件,努力扩大普通高中和职业高中规模。搞好新城区教育布局,加快区内中小学建设。完成企业分离学校任务。不断提高高等教育质量,办出我市高等教育特色。加快发展文化卫生体育事业。开工建设市博物馆、市文化艺术中心。努力实现县县都有功能齐全、管理规范的图书馆、文化馆。完善乡镇综合文化站、行政村文化活动室,健全农村文化服务网络,繁荣城乡文化事业。切实搞好文物和民族民间文化遗产保护,并合理开发利用。扩大农村广播电视和信息网络覆盖面,实施居民20户以上的自然村广播电视“村村通”工程。加强医疗卫生基础设施建设,市第一人民医院病房楼、第二人民医院心血管病房楼、妇幼保健院产科大楼年内投用,平煤总医院综合门诊楼部分投用。筹措资金1359万元,完成乡镇卫生院改造任务。健全社区卫生服务网络,努力为居民提供安全、便捷、经济的公共卫生和基本医疗服务。积极发展体育事业,广泛开展群众性体育活动,精心组织好省十运会参赛工作。同时,深化人口和计划生育综合改革,充实完善考核办法,稳定低生育水平,提高出生人口素质,优化出生人口结构,确保完成年度工作任务。办好爱心救助超市,做好经常化、社会化捐赠救助工作。加强基层统计建设,搞好第二次全国农业普查。

(八)认真解决关系人民群众切身利益的问题。千方百计扩大就业。筹措资金6000万元,支持下岗职工再就业2.4万人,其中“4050”人员8800人。认真落实就业再就业优惠政策,多渠道开发就业岗位,确保新增就业6万人。进一步完善社会保障体系。继续扩大城镇社会保险覆盖面,认真解决进城务工人员、非公有制企业从业人员的社会保障问题。完善城镇职工基本养老保险制度,确保离退休人员基本养老金按时足额发放。提高城镇居民最低生活保障标准。加强城镇基本医疗保险和失业、工伤、生育保险制度建设。做好住房公积金管理工作,为城镇职工购(建)房提供贷款支持。全面建立农村新型合作医疗制度。筹措资金1600万元,全面实行农村最低生活保障。抓好农村敬老院建设,提高“五保户”集中供养率。继续落实好农村计划生育家庭的各项优惠扶持政策。努力提高居民收入和生活质量。严格执行最低工资制度。改革公务员工资制度,规范津贴、补贴。提高城镇中低收入者的收入水平。认真清理解决建设领域拖欠工程款和农民工工资问题,坚决防止出现新的拖欠。进一步整顿和规范市场经济秩序,严厉打击制售假冒伪劣产品、商业欺诈等违法犯罪活动。加强食品药品安全监管。切实加强安全生产。认真执行安全生产的各项法律法规,全面落实各级安全生产责任制。始终保持清醒头脑和高度警惕,对安全生产任何时候都不能有丝毫的麻痹和松懈。进一步加强领导,完善机制,加大投入,强化监管,做好地方煤矿、非煤矿山、道路交通、民爆物品、消防、建筑、危险化学品、烟花爆竹等重点行业、重点领域的安全工作,严厉打击各类违法违规生产,坚决遏制重特大事故的发生,确保人民群众的生命财产安全。全力维护社会稳定。强化社会治安综合治理,完善社会治安防控体系,严厉打击各类犯罪活动,切实增强人民群众的安全感。严密防范境外敌对势力、邪教组织和各种暴力恐怖活动,维护国家安全,建设平安鹰城。继续做好人民群众来信来访工作,认真排查调处不稳定因素,积极预防和妥善处置群体性事件。

(九)切实加强社会主义精神文明和民主法制建设。大力弘扬焦裕禄精神、红旗渠精神和愚公移山精神。继续加强公民道德建设。不断改进青少年思想道德建设以及大学生思想政治教育工作。繁荣发展新闻出版、广播电视、文化艺术和哲学社会科学。扎实推进文明城市、文明景区、文明社区、文明村镇、文明村组、文明家庭等群众性精神文明创建活动,组织好文化、科技、卫生“三下乡”和科技、文体、法律、卫生“四进社区”活动。深入开展普法教育,提高全民法律素质。进一步提高政府工作法制化水平,自觉接受人大及其常委会的法律监督、工作监督和人民政协的民主监督,严格执行人大及其常委会的决议、决定,支持人大代表依法行使职权,认真办理人大代表建议和政协提案。建立市政府与市人大、市政协的联系沟通制度,广泛听取各民主党派、工商联、无党派代表人士的意见和建议,充分发挥工会、共青团、妇联等人民团体的桥梁纽带作用。全面贯彻党的民族宗教政策,做好民族宗教工作。加强法律援助工作,依法保障妇女、未成年人和残疾人的合法权益。继续做好技术监督、盐业、史志、对台、侨务、档案、气象、地震、老龄等工作。

积极支持部队和国防建设。深入开展国防教育,健全国防动员体系。关心和支持驻平部队建设,抓好人民防空和民兵预备役工作。认真落实军人优待抚恤和退伍安置政策,继续深入开展“双拥”活动,再创全国“双拥模范城”。

(十)努力建设法治政府、诚信政府和服务型政府。近年来,我市在行政管理体制改革方面虽然取得了一定成效,政府职能和工作作风有了较大转变,行政效能和服务水平有了明显提高,但从群众反映的情况看,一些地方和部门开拓创新不够,服务大局意识不强,工作效率不高,“四乱”现象、吃拿卡要、官僚主义、形式主义依然存在,离人民群众的期望和投资业主的要求还有一定差距。为了从制度上解决问题,去年,市政府通过几个月调研,在反复征求各方面意见的基础上,出台了《平顶山市人民政府关于建设法治诚信服务型政府的意见》(以下简称《意见》)。今年加强政府自身建设的主要任务,就是认真落实《意见》要求,确保取得明显成效。加快建设法治政府。强力推行行政执法责任制,进一步理顺部门分工,明确职能、权限和责任。认真贯彻行政许可法和依法行政实施纲要,健全对行政管理权力的监督机制。加强行政法制建设和经济法制建设,建立权责明确、行为规范的行政执法体制,落实行政执法主体资格制度,保证各级行政管理机关及其工作人员严格按照法定权限和程序行使职权、履行职责。完善行政管理决策机制,健全重大事项决策的协商和协调机制,健全对专业性、技术性较强的重大事项决策的专家论证、技术咨询、决策评估制度,健全对群众利益密切相关的重大事项决策的公示、听证制度,推进行政管理决策科学化、民主化。建立体现科学发展观和正确政绩观要求的干部实绩考核评价制度,完善政府责任目标考评体系,增加衡量质量效益、和谐社会建设等方面的指标,实施GDP能耗指标公报制度。健全对公务员廉洁从政的监督约束机制,全面推行领导干部经济责任审计和离任监察制度。集中开展治理商业贿赂专项工作。落实廉政建设责任制,加大对腐败渎职行为的惩治力度。加快建设诚信政府。坚持合法合理和公正公平原则,平等对待行政管理相对人,制定的政策文件和作出的决策决定,非因法定事由并经法定程序不得改变,保持政策和决定的稳定性。继续推行服务承诺,大力整肃信用秩序,建立行政机关违反诚信原则的责任追究制度,做到言必信、行必果,取信于民。加快建设服务型政府。改进政府管理方式,创新政府管理机制,实行政企分开、政资分开、政事分开、政府与市场中介组织分开。搞好经济调节和市场监管,着力为市场主体创造公平竞争环境和提供良好服务;更加注重履行社会管理和公共服务职能,把政府工作的着力点更多地放在为经济发展创造良好环境、发展社会事业和构建和谐社会上。推进政务公开,在公共服务部门和单位全面推行办事公开制度。积极整合行政资源,有效降低行政成本。健全行政服务大厅功能,继续精减办事程序,切实搞好“一站式”服务。办好市长热线,认真负责地办理群众反映的问题。建立健全社会预警体系和应急救援、社会动员机制,提高处置自然灾害、安全事故、动物疫情、公共卫生、重大环境污染等突发事件和保障公共安全的能力。

各级政府和政府各部门必须按照《意见》要求,制定出既能操作又能检查考核的目标,并认真组织落实。市政府推进法治、诚信和服务型政府建设工作领导小组及办公室要切实负起责任,加强指导和督促检查。市长、市政府领导班子、市政府各部门的主要负责同志要起带头和表率作用。同时,以贯彻实施《公务员法》为契机,进一步加强对公务员的教育、管理和监督,使广大公务员忠于职守、勤勉敬业、遵纪守法,在人民群众中树立良好形象。

最近,党中央、国务院明确提出了建设创新型国家的目标和任务,这是落实科学发展观的又一重要战略决策。我们在加快建设法治政府、诚信政府和服务型政府的同时,要努力建设创新型政府,带动建设创新型城市。通过强化政府创新、企业创新、社会创新,促进我市经济社会更快更好发展。

各位代表,平顶山是一座充满生机与希望的城市,我们肩负的责任重大而光荣,我们面临的任务艰巨而繁重。让我们在省委、省政府和市委的坚强领导下,开拓创新,扎实苦干,团结拼搏,全面实现今年的各项工作目标,为“十一五”发展开好局、起好步。
